# Noises in the Ears

**Published:** 1890-03-29

**Authors:** 


### NOISES IN THE EARS.

Dr. Kiesselbach recommends for this frequent and annoying symptom the injection of 10 drops of a 4—10 per cent. solution of cocaine at intervals of three to five days through an ear speculum or tube. Occasionally a feeling of giddiness or even vomiting may follow the first injection, but this does not recur later. The first injection should, therefore, be of the weaker solution, and the strength gradually increased.

